# Acute tubular nephropathy in a patient with acute HIV infection: review of the literature

**DOI:** 10.1186/1742-6405-11-34

**Published:** 2014-11-07

**Authors:** Jintanat Ananworanich, Anandita A Datta, James LK Fletcher, Natavudh Townamchai, Nitiya Chomchey, Eugene Kroon, Irini Sereti, Victor Valcour, Jerome H Kim

**Affiliations:** SEARCH, The Thai Red Cross AIDS Research Centre, Bangkok, Thailand; Department of Medicine, Faculty of Medicine, Chulalongkorn University, 1873 Rama IV, Pathumwan, Bangkok 10330 Thailand; US Military HIV Research Program, Walter Reed Army Institute of Research, 503 Robert Grant Avenue, Silver Spring, MD 20910 USA; US Military HIV Research Program, Henry M. Jackson Foundation for the Advancement of Military Medicine, 6720A Rockledge Drive, Suite 400, Bethesda, MD 20817 USA; Division of Nephropathology, Joint Pathology Center, 606 Stephen Sitter Avenue, Silver Spring, MD 20910 USA; Clinical and Molecular Retrovirology Section/ Laboratory of Immunoregulation, 10 Center Drive, building 10, room 11B-07A, Bethesda, MD 20892 USA; Memory and Aging Center, University of California at San Francisco, Suite 905, 350 Parnassus Avenue, San Francisco, CA 94143 USA

**Keywords:** HIV, Acute renal failure, Acute tubular nephropathy, Acute retroviral syndrome, Acute HIV infection, Elderly

## Abstract

We report a 57-year old man with diabetes mellitus and hypertension who presented with acute HIV infection. Routine blood tests showed an elevated blood urea nitrogen and creatinine. Renal biopsy showed acute tubular nephropathy, which has not been reported to occur during acute HIV infection, in the absence of rhabdomyolysis or multiple organ system failure. Antiretroviral therapy was initiated. His renal failure gradually resolved without further intervention. At one year of follow-up his HIV RNA was undetectable, and his renal function was normal. The case illustrates a rare manifestation of acute HIV infection – acute renal failure - in an older man with diabetes and hypertension. In this setting acute kidney injury might mistakenly have been attributed to his chronic comorbidities, and this case supports early HIV-1 testing in the setting of a high index of suspicion.

## Background

Renal diseases afflicting persons living with HIV are almost always chronic in nature and occur late in the course of illness. Chronic renal diseases occur in up to 10% of these individuals, and older age, low CD4 count and use of nephrotoxic antiretrovirals are major predictive factors [1, 2]. Acute renal failure (ARF) during acute or primary HIV infection is exceedingly rare, and has been reported in the setting of rhabdomyolysis or multi-organ system failure [3–7]. The pathogenesis is not well understood but is thought to be from the rapid rise of HIV viremia and inflammation associated with acute HIV infection (AHI) [8, 9].

We here report an older man with diabetes and hypertension who was identified in an AHI screening study and was found to have ARF. Supportive care of ARF and treatment of AHI were associated with normal renal function and successful suppression of HIV-1 at one year of follow-up.

## Case presentation

A 57-year old Thai man presented for HIV screening at an anonymous testing center in Bangkok where routine evaluation for AHI is performed [10]. At presentation, the patient was reactive to the HIV antigen-antibody combination 4th generation enzyme immunoassay (EIA, AxSYM, Abbott Laboratories, Wiesbaden, Germany) and the IgM-sensitive 3rd generation HIV EIA (Genscreen HIV 1/2, Bio-Rad, Marnes la Coquette, France) but was non-reactive to the IgG-sensitive 2nd generation HIV EIA (Genetic Systems rLAV EIA, BioRad Laboratories, Redmond, WA). This signified infection within the past 4 weeks [10], and consistent with his history of HIV exposure via unprotected sex with a female sex worker 30 days prior. Twelve days prior to AHI diagnosis, he experienced symptoms consistent with acute retroviral syndrome (fever, fatigue, diarrhea, nausea and vomiting) and was hospitalized for 3 days for intravenous hydration; no laboratory tests are available from that admission. At the time of presentation with AHI diagnosis, he reported only mild fatigue, and did not have oliguria or edema. Blood pressure was 174/99 mmHg, pulse rate was 62 beats/minute and temperature was 37.2 C. His HIV RNA was 81,515 copies/ml and CD4 was 313 cells/mm^3^. Three days later, he consented to enrollment in an AHI study, by which time his HIV RNA was 354,706 copies/ml. However the 2nd generation HIV EIA remained non-reactive. Routine clinical chemistries revealed a creatinine of 8 mg/dl and an estimated glomerular filtration rate (eGFR) of 7.8 ml/min/1.73 m^2^ using the Modification of Diet in Renal Disease formula corrected for Thai ethnicity [11]. Abnormalities were confirmed by subsequent sampling. Urinalysis showed a specific gravity 1.010, pH 5.0, 1+ proteinuria, 1+ leukocytes, 1+ blood, negative glucose, ketone, and bilirubin, 3–5 white blood cells/HPF, 2–3 red blood cells/HPF, 1–2 epithelial cells/HPF and no casts. Spot urine was tested for protein/creatinine ratio (0.14) and fractional excretion of sodium (2.47%). Other clinical laboratories included: hemoglobin 15.5 g/dl, alanine transaminase 81 U/l, creatine phosphokinase 54 U/L, uric acid 12 mg/dl, calcium 8.4 mg/dl, phosphate 5.5 mg/dl, albumin 3.7 g/dl, sodium 132 mmol/l, potassium 4.4 mmol/l, chloride 97 mmol/l and carbon dioxide 21 mmol/l. Fasting blood glucose (135 mg/dl) and HbA1C (8.2%) were elevated. Hepatitis B surface antigen, anti-hepatitis C antibody and rapid plasma reagent for syphilis were negative. The Epstein Barr virus (EBV) IgM was negative at time of ARF and 4 weeks later while IgG levels were 76 U/ml and 86 U/ml, respectively. Cytomegalovirus (CMV) IgM was weakly positive (level of 0.28, cut off 0.19 units) at baseline and negative 4 weeks later while IgG levels were 155 RU/ml and 191 RU/ml at these 2 time points.

His past history included hypertension and diabetes mellitus (DM) diagnosed 4 years prior, and he was treated with once daily oral metformin (500 mg), glyburide (5 mg), atenolol (100 mg), amlodipine (10 mg). Six months before AHI, his BUN and creatinine were 25 and 1.8 mg/dl with no proteinuria and trace glucosuria. The patient denied taking non-steroidal anti-inflammatory or other nephrotoxic drugs. He complained of intermittent episodes of palpitation during AHI, captured by cardiac monitoring during this hospitalization for ARF and characterized as atrial fibrillation that recovered spontaneously.

**Figure 1:**
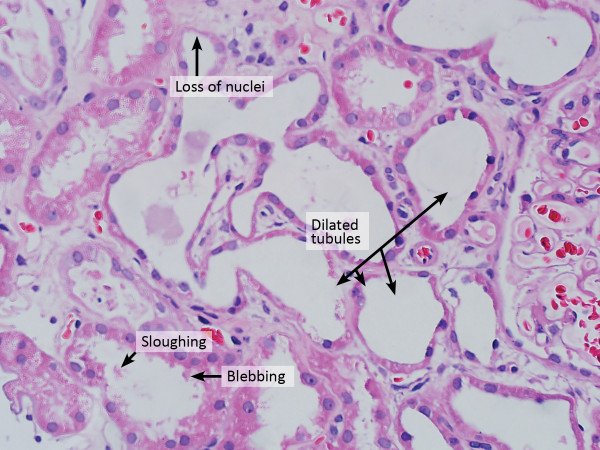
**Light microscope (Hematoxylin Eosin stain) of the kidney tissue showed dilated tubules, focal epithelial attenuation, blebbing, sloughing and nuclear dropout.**

**Figure 2:**
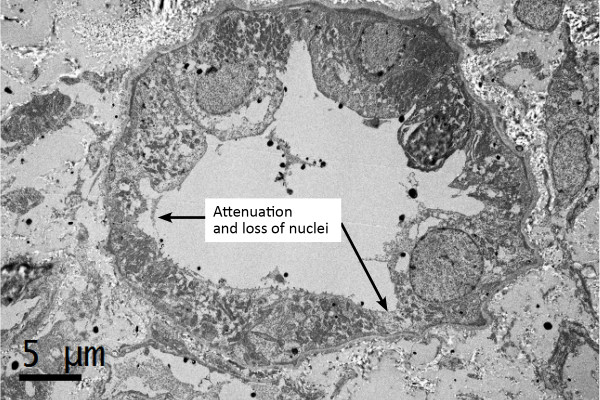
**Electron microscope of the kidney tissue showed attenuation and loss of nuclei.**

Renal ultrasonography demonstrated normal size and echogenicity of both kidneys with no apparent structural abnormalities. Renal biopsy identified tubular changes consistent with acute tubular nephropathy. By light microscopy, some tubules were dilated withfocal epithelial attenuation, blebbing, sloughing and nuclear dropout (Figure 1). Electron microscopic examination revealed attenuation and loss of nuclei are observed (Figure 2). Loss of the brush border was seen in proximal tubules. There was moderate interstitial fibrosis associated with a scattered inflammatory cell infiltration of mainly mononuclear cells and a few eosinophils. Glomeruli were normocellular. There was no evidence of immune complexes, fibrinoid necrosis or wire-loop lesions. Viral inclusions were not identified in tubular epithelium on electron microscopy. Arteries and arterioles were moderately thickened.

The patient received supportive care without dialysis in the hospital for 2 weeks and his eGFR gradually rose to 30 mg/min/1.73 m^2^ at week 2 following AHI diagnosis. Antiretroviral therapy (ART) was initiated on day 11 with eGFR-adjusted dosages of zidovudine and lamivudine, given in combination with standard dosage of efavirenz. His HIV RNA declined rapidly to 446 copies/ml at week 4 of ART. After 12 weeks of ART, his HIV RNA declined to 72 copies/ml, the CD4 rose to 445 cells/mm^3^, and his renal function had normalized with a creatinine of 1.0 mg/dl, eGFR of 85 ml/min/1.73 m^2^. Repeat urinalysis was normal. His fasting glucose was 144 mg/dl and blood pressure was 121/108 mmHg on the same medications for DM and hypertension. At one year following the ARF episode, he had an undetectable HIV RNA, CD4 of 618 cells/mm^3^, creatinine of 1.1 mg/dl, eGFR of 77 ml/min/1.73 m^2^ and normal urinalysis. He is currently doing well except for occasional episodes of palpitations precipitated by fatigue.

### Literature review

We searched the Pubmed database with the following key words: primary HIV infection, AHI, ARF, acute tubular nephropathy, acute tubular necrosis, multiple organ failure and rhabdomyolysis to identify case reports published in the English-language literature from January 1984 to July 2014. We included only cases with AHI/primary HIV infection and creatinine abnormalities of grade 2 and above (≥1.8 mg/dl) according to the US Department of Health and Human Services. AHI is defined by having a positive HIV nucleic acid and/or reactive IgM-sensitive HIV antibody testing with non-reactive IgG-sensitive HIV antibody testing and/or Western Blot [10, 12]. Primary HIV infection is defined as HIV seroconversion within the past 6 months.

There were 7 reported cases (Table 1). In 4 cases, nephropathy occurred as part of rhabdomyolysis [2, 5–7], 1 had hemolytic uremic syndrome (HUS) [3], and 2 cases had HIV-associated nephropathy (HIVAN) [1, 4].Three of 4 patients with rhabdomyolysis had acute tubular necrosis by renal biopsy and 1 case had renal infarcts by CT scan. HIVAN, a sclerosing glomerulopathy, is associated with advanced HIV infection, but in both patients reported here, it occurred during the period of high HIV RNA and prior to HIV seroconversion [1, 4] HUS, another complication usually seen in advanced HIV infection, was reported in a case with high HIV viremia associated with AHI [3]. The serum creatinine ranged from 2.3 to 8 mg/dl, and 4 of 7 patients required hemodialysis.

**Table 1:** **Published cases of acute renal failure during acute/primary HIV infection**

First author (year)	Age, race, gender	Acute/primary HIV infection diagnosis	Renal disease diagnosis	Other findings	Outcome of acute renal failure
del Rio C (1990) [2]	29-year old, African American man	Non-reactive HIV EIA and Western Blot with seroconversion 4 weeks after presentation	Acute tubular necrosis and mesangioproliferative glomerulonephritis by renal biopsy, Creatinine was 2.8 mg/dl and 24-hour urine protein was 6 g	Rhabdomyolysis	Improved after supportive care
Levin ML (2001) [4]	41-year old, Black male	Non-reactive HIV EIA with HIV RNA 700,000 copies/ml. Patient experienced HIV seroconversion 6 weeks later	Acute renal failure from HIVAN diagnosed by renal biopsy. Creatinine was 7.3 mg/dl and 24-hour urine protein was 6.9 g.	None reported	Improved with supportive care and antiretroviral therapy
Prabahar MR (2008) [7]	42-year old, Saudi male	Reactive HIV EIA but primary HIV infection was presumed because of high HIV RNA and consistent clinical profile	Acute renal tubular necrosis diagnosed by renal biopsy with a creatinine of 6.8 mg/dl	Rhabdomyolysis, acute hepatitis	Improved after hemodialysis
Pano-Pardo JR (2009) [6]	19-year old, Haitian American female	HIV seroconversion within the past 8 weeks	Bilateral renal infarcts on CT scan, acute renal failure with a creatinine of 2.3 mg/dl	Rhabdomyolysis, myocarditis, pancreatitis, anemia	Recovered with supportive care
Gomes AM (2009) [3]	38-year old, Black male	Inconclusive HIV EIA and non-reactive Western Blot with HIV RNA >1,000,000 copies/ml	Acute renal failure with a creatinine of 7.5 mg/dl. Renal biopsy was not done due to risk of bleeding	Hemolytic uremic syndrome (hemolytic anemia, thrombocytopenia)	Improved after plasmapheresis and hemodialysis
Merrill ER (2011) [5]	19-year old, African American male	HIV seroconversion during the hospitalization period	Acute tubular necrosis diagnosed by renal biopsy with a creatinine of 8 mg/dl and abnormal urinalysis (3+ protein, 3+ blood, 158 red cells/HPF)	Rhabdomyolysis, acute hepatitis. CMV IgG was positive	Improved after hemodialysis
Szabo S (2002) [1]	47-year old, African American woman	Reactive HIV EIA and a negative Western Blot with HIV RNA >75,000 copies/ml	Creatinine of 14 mg/dl and 24-hour urine protein of 21.4 g. Renal biopsy showed severe collapsing focal segmental glomerulosclerosis consistent with HIVAN	None reported	Improved after hemodialysis but later died from bacterial sepsis

Footnote: HIVAN: HIV-associated nephropathy, EIA: enzyme immunoassay, CMV: Cytomegalo virus.

## Discussion

Our patient illustrates a rare manifestation of ARF associated with AHI. There are several important clinical points. Firstly, the patient is an older man, thus in a demographic group where HIV may not have been suspected should he have presented for clinical care due to renal findings. Recently, however, older individuals are emerging as an important at-risk group for HIV [13], and patients’ age should not deter health care personnel from obtaining history of possible exposure to HIV, and performing HIV testing when indicated. Secondly, his DM and hypertension may have been mistaken as the sole cause for his ARF, missing the opportunity to diagnose HIV infection. It is likely that some degree of chronic renal insufficiency from these co-morbidities was already present in our patient, predisposing him to ARF when he became acutely infected with HIV. Such co-morbidities are increasing in the general population, particularly among elders [14]. Together with the rising HIV epidemic in this age group, we may be seeing more cases of ARF from AHI, and awareness of such condition by health care personnel is critical. The renal pathology in our patient is not consistent with DM, hypertension, or autoimmune diseases, and we did not find other causes by history and laboratory testing for rhabdomyolysis and common viral infections.

The pathogenesis of acute tubular nephropathy in AHI is unclear but in the case of HIVAN, it is thought to be due to the rapid rise in HIV viremia resulting in a burst of uncontrolled systemic inflammation, sometimes called "the cytokine storm" [9], which in turn could cause activation of local inflammatory mediators [8]. In our patient with a milder form of disease, direct HIV infection of tubular cells could be a possible cause. Among 19 HIV-infected patients with end-stage renal disease who underwent kidney transplantation, 62% had HIV detected in the tubular cells of the kidney without podocyte infection [15]. Renal pathology of acute tubular nephropathy from any cause generally shows tubular abnormalities as seen in our patient (see Table 1). Additional causes of acute tubular nephropathy may include autoimmune diseases or other viral infections such as hepatitis B and C, CMV, EBV, and they should be excluded. It is possible that our patient had a recent CMV infection as he had a weakly positive CMV IgM titer at presentation; however, there was no demonstrable rise in CMV IgG titer 4 weeks later. Transient ischemia from atrial fibrillation may have contributed to the ARF. Clinical symptoms of acute tubular nephropathy could be severe with oliguria and uremia. We did not observe this in our patient likely because he was diagnosed early with AHI. Treatment includes supportive care and ART to reduce HIV plasma viremia and reverse inflammation. Renal adjustment of ART dosing according to eGFR is required. Fortunately, with proper diagnosis and treatment, the condition generally recovers.

## Conclusion

Acute tubular nephropathy can occur in patients with AHI. Patients who have underlying renal insufficiency including older patients and those with co-morbidities may be at greater risk. HIV testing should be performed, and in cases with a high index of suspicion, a negative HIV antibody test should be followed by a sensitive HIV immunoassay such as the 4th generation assay or nucleic acid testing to facilitate prompt diagnosis and management.

## Consent

Patient provided written consent for his participation in the acute HIV infection study for which information was recorded.

## Abbreviations

AHI: Acute HIV infection
ARF: Acute renal failure
ART: Antiretroviral therapy
CMV: Cytomegalo virus
DM: Diabetes mellitus
EIA: Enzyme immuneassay
eGFR: Estimated glomerular filtration rate
EBV: Epstein Barr virus
HIVAN: HIV-associated nephropathy.

## References

[1] Szabo S, James CW, Telford G (2002). Unusual presentations of primary human immunodeficiency virus infection. AIDS Patient Care STDS.

[2] del Rio C, Soffer O, Widell JL, Judd RL, Slade BA (1990). Acute human immunodeficiency virus infection temporally associated with rhabdomyolysis, acute renal failure, and nephrosis. Rev Infect Dis.

[3] Gomes AM, Ventura A, Almeida C, Correia M, Tavares V, Mota M, Seabra J (2009). Hemolytic uremic syndrome as a primary manifestation of acute human immunodeficiency virus infection. Clin Nephrol.

[4] Levin ML, Palella F, Shah S, Lerma E, Butter J, Kanwar YS (2001). Hiv-associated nephropathy occurring before HIV antibody seroconversion. Am J Kidney Dis.

[5] Merrill E (2011). Acute HIV rhabdomyolysis, renal failure, and hepatitis: A case report. The Journal of Thomas Jefferson University Hospital, Department of Internal Medicine.

[6] Pano-Pardo JR, Alcaide ML, Abbo L, Dickinson G (2009). Primary HIV infection with multisystemic presentation. Int J Infect Dis.

[7] Prabahar MR, Jain M, Chandrasekaran V, Indhumathi E, Soundararajan P (2008). Primary HIV infection presenting as non-traumatic rhabdomyolysis with acute renal failure. Saudi J Kidney Dis Transpl.

[8] Abitbol CL, Friedman LB, Zilleruelo G (2005). Renal manifestations of sexually transmitted diseases: sexually transmitted diseases and the kidney. Adolesc Med Clin.

[9] Stacey AR, Norris PJ, Qin L, Haygreen EA, Taylor E, Heitman J, Lebedeva M, DeCamp A, Li D, Grove D, Self SG, Borrow P (2009). Induction of a striking systemic cytokine cascade prior to peak viremia in acute human immunodeficiency virus type 1 infection, in contrast to more modest and delayed responses in acute hepatitis B and C virus infections. J Virol.

[10] Ananworanich J, Fletcher JL, Pinyakorn S, van Griensven F, Vandergeeten C, Schuetz A, Pankam T, Trichavaroj R, Akapirat S, Chomchey N, Phanuphak P, Chomont N, Michael NL, Kim JH, de Souza M (2013). A novel acute HIV infection staging system based on 4th generation immunoassay. Retrovirology.

[11] Praditpornsilpa K, Avihingsanon A, Chaiwatanarat T, Chaiyahong P, Wongsabut J, Ubolyam S, Chulakadabba A, Avihingsanon Y, Ruxrungtham K, Tunsanga K, Eiam-Ong S, Phanuphak P (2012). Comparisons between validated estimated glomerular filtration rate equations and isotopic glomerular filtration rate in HIV patients. AIDS.

[12] Ananworanich J, Schuetz A, Vandergeeten C, Sereti I, de Souza M, Rerknimitr R, Dewar R, Marovich M, van Griensven F, Sekaly R, Pinyakorn S, Phanuphak N, Trichavaroj R, Rutvisuttinunt W, Chomchey N, Paris R, Peel S, Valcour V, Maldarelli F, Chomont N, Michael N, Phanuphak P, Kim JH (2012). Impact of multi-targeted antiretroviral treatment on gut T cell depletion and HIV reservoir seeding during acute HIV infection. PLoS One.

[13] Metallidis S, Tsachouridou O, Skoura L, Zebekakis P, Chrysanthidis T, Pilalas D, Bakaimi I, Kollaras P, Germanidis G, Tsiara A, Galanos A, Malisiovas N, Nikolaidis P (2013). Older HIV-infected patients-an underestimated population in northern Greece: epidemiology, risk of disease progression and death. Int J Infect Dis.

[14] Reuter S, Oette M, Kaiser R, Lengauer T, Fatkenheuer G, Rockstroh JK, Knechten H, Haussinger D (2012). Risk factors associated with older age in treatment-naive HIV-positive patients. Intervirology.

[15] Canaud G, Dejucq-Rainsford N, Avettand-Fenoel V, Viard JP, Anglicheau D, Bienaime F, Muorah M, Galmiche L, Gribouval O, Noel LH, Satie AP, Martinez F, Sberro-Soussan R, Scemla A, Gubler MC, Friedlander G, Antignac C, Timsit MO, Onetti Muda A, Terzi F, Rouzioux C, Legendre C (2014). The kidney as a reservoir for HIV-1 after renal transplantation. J Am Soc Nephrol.

